# Sex differences in foetal origins of child emotional symptoms: a test of evolutionary hypotheses in a large, general population cohort

**DOI:** 10.1111/jcpp.13229

**Published:** 2020-03-20

**Authors:** Elizabeth C. Braithwaite, Andrew Pickles, Nicola Wright, Helen Sharp, Jonathan Hill

**Affiliations:** ^1^ Department of Psychology Faculty of Health, Psychology and Social Care Manchester Metropolitan University Manchester UK; ^2^ Department of Biostatistics and Health Informatics Institute of Psychiatry, Psychology and Neuroscience King's College London London UK; ^3^ Department of Psychological Sciences Faculty of Health and Life Sciences University of Liverpool Liverpool UK; ^4^ School for Psychology and Clinical Language Sciences University of Reading Reading UK

**Keywords:** Sex differences, postnatal, maternal depression, prenatal, emotional disorder

## Abstract

**Background:**

Based on previous findings from the Wirral Child Health and Development Study (WCHADS), and on evolutionary hypotheses, we preregistered analyses of data from a large epidemiological sample (https://osf.io/fn5g9/register/564d31db8c5e4a7c9694b2be), to test for sex‐dependent moderation by prenatal maternal depressive symptoms of the association between postnatal maternal depressive symptoms and child emotional problems.

**Methods:**

A total of 8,354 mothers and children were followed from pregnancy to 3.5 years in the Avon Longitudinal Study of Parents and Children (ALSPAC). Self‐report measures of prenatal and postnatal maternal depressive symptoms, and maternal report of child emotional symptoms were administered.

**Results:**

There was a three‐way interaction between maternal prenatal and postnatal depression, and child sex (Coeff .042 95% CI 0.015 to 0.068, *p* = .002). This arose from moderation by prenatal depression, in opposite directions in boys and in girls. In boys, the association between postnatal depression and child emotional symptoms was weaker following lower prenatal depressive symptoms (interaction term coeff = .030, *p* = .001), and in girls, to a lesser extent, the association was stronger following lower prenatal depressive symptoms (interaction term coeff = −.012, *p* = .221).

**Conclusions:**

We replicated the finding from the WCHADS that prenatal depression modifies the association between postnatal depression and children's emotional problems in a sex‐dependent fashion. In ALSPAC, the sex difference was explained mainly by a protective effect of low prenatal depression in boys, while in WCHADS, it arose from greater vulnerability of girls to postnatal depression following low prenatal depression. In the light of these findings, in evaluating and implementing early interventions, there is need to consider that risks associated with postnatal depression may vary depending on maternal mood during pregnancy and may differ between boys and girls.

## Introduction

According to the ‘foetal origins’ hypothesis, foetal adaptations occur *in utero* in anticipation of later environmental exposures (Barker, 2007). This theory was first proposed to account for associations between low birth weight and later obesity, cardiovascular disease and type II diabetes, whereby low birthweight reflects an evolved adaptive mechanism that confers advantages in later environments of food scarcity, but simultaneously creates risk in the presence of a high‐calorie diet. It is evident, however, that adaptations prior to birth that anticipate later environments are found across species, reflecting a conserved ‘Predictive Adaptive Response’ (PAR) mechanism (Bateson, Gluckman & Hanson, 2014; Wells, 2007). More generally, it is hypothesised that matched prenatal and postnatal environments will be associated with positive offspring outcomes, whereas a mismatch between the prenatal and postnatal environment confers risk for poor outcomes.

There is also evidence that foetal adaptations to *in utero* environmental conditions vary by sex. The Trivers–Willard (T‐W) hypothesis frames this difference within an evolutionary context based on reproductive fitness (Trivers & Willard, 1973). According to the T‐W hypothesis, if maternal health during pregnancy predicts later offspring reproductive fitness, then mothers in good health will ‘invest’ in males leading to more male than female births, because healthy males compete more successfully for females. Alternatively, when maternal conditions are poor, investment in females is favoured and the sex ratio is reversed, to avoid bearing unsuccessful males. This hypothesis is consistent with well‐documented observations that male foetuses are more vulnerable than females to threats, such as preterm birth and maternal asthma, and are also more likely to suffer neurodevelopmental consequences of foetal insults (Aiken & Ozanne, 2013).

The prediction from the two hypotheses jointly is that if foetal adaptations anticipate later environments, and the female foetus is more able than the male to adapt to poor maternal conditions, then we expect female offspring of mothers with poor conditions during pregnancy later exposed to adverse environments to have better outcomes than males who have been similarly exposed. Conversely, female offspring who are not exposed to poor maternal conditions during pregnancy are less well prepared and hence have poorer outcomes compared to males, when exposed to a negative environment.

If these mechanisms apply to prenatal and postnatal exposures to maternal mood, and to behavioural outcomes, the implication is that, in addition to independent contributions of prenatal and postnatal depression or anxiety, their interaction may also influence children's outcomes. This has not been tested formally using moderation models, prior to publications from WCHADS. However, the predictions based on the T‐W and PAR hypotheses have been examined comparing matched prenatal and postnatal depression groups (low–low and high–high) with mismatched groups (low–high and high–low; Sandman, Glynn & Davis, 2013). Prenatal–postnatal matching was associated with better cognitive and motor outcomes over the first year of life than mismatching, and at 6 months, this effect was only evident in females (Sandman, Glynn & Davis, 2013). In analyses of data from the WCAHDS, prenatal maternal depressive symptoms modified the association between postnatal depression and child anxious‐depressed symptoms as predicted by the PAR hypothesis, and in a sex‐dependent manner as predicted by the T‐W hypothesis. In girls, the association between postnatal depressive symptoms and child anxious‐depressed symptoms was stronger among those exposed to lower prenatal maternal depression, in contrast to those exposed to higher levels (Hill et al., 2017). Furthermore, this effect was mediated via *NR3C1* methylation levels at 14 months (Hill, Pickles, Wright, Quinn, et al., 2019). This mismatch effect was not seen in males, and the sex difference was supported by a three‐way interaction between prenatal and postnatal depression and child sex. We have also shown moderation by prenatal anxiety of the association between postnatal maternal anxiety and child irritability at age 7 years, further moderated by levels of maternal stroking during infancy (Hill, Pickles, Wright, Braithwaite & Sharp, 2019). This effect also was seen only in girls.

The aim of the current study was to replicate the sex‐dependent moderator effect of prenatal depression on the association between postnatal depression and child emotional symptoms found in the WCHADS study, using data from a larger birth cohort: the Avon Longitudinal Study of Parents and Children (ALSPAC). We preregistered the hypothesis that there will be a three‐way interaction between prenatal and postnatal maternal depressive symptoms and child sex in the prediction of children's emotional symptoms at age 3.5 years (Braithwaite, 2018). We also predicted that this would arise from a stronger association between postnatal depression and child symptoms following low, contrasted with high, levels of prenatal depression, and seen only in girls. We further planned and preregistered exploratory analyses to examine timing effects of prenatal depression (second vs third trimester) and to test whether similar effects were evident for maternal anxiety.

## Methods

### Participants and procedure

The present study is an analysis of data collected as part of the Avon Longitudinal Study of Parents and Children (ALSPAC). ALSPAC is a large, population‐based longitudinal study in which pregnant women resident in Avon, UK, with expected dates of delivery 1 April 1991 to 31 December 1992 (Boyd et al., 2013; Fraser et al., 2013) were invited to take part in the study. The initial number of pregnancies enrolled was 14,541. Of these initial pregnancies, there was a total of 14,676 foetuses, resulting in 14,062 live births and 13,988 children who were alive at 1 year of age. Questionnaires were sent to parents at regular intervals during pregnancy and after childbirth.

The WCHADS report, on which the preregistration was based, made use of maternal reports of depressive symptoms at 20 weeks gestation, and 9 weeks, 7 months and 14 months after birth. In ALSPAC, maternal depressive symptoms were available at 18 weeks gestation, and at 8 weeks, 8 months and 21 months after birth. In WCHADS, maternal reports of child emotional difficulties at ages 2.5, 3.5 and 5 years were available and in ALSPAC at age 3.5 years. Variables included as confounders were reported by mothers during pregnancy, as they were for the WCHADS sample. For the planned and preregistered exploratory analyses, respectively, we used maternal reports of prenatal depression at 32 weeks gestation (to test for prenatal timing effects) and maternal reports of prenatal anxiety at 18 and 32 weeks gestation, and at 8 weeks, 8 months and 21 months after birth (to examine specificity to depression).

#### Ethical considerations

Ethical approval was obtained from the ALSPAC law and ethics committee, and from local research and ethics committees. Informed consent for the use of data collected via questionnaires and clinics was obtained from participants following the recommendations of the ALSPAC Ethics and Law Committee at the time.

### Measures

#### Maternal depression

Mothers self‐reported symptoms of depression during pregnancy (18 and 32 weeks gestation) and during the postnatal period (8 weeks, 8 months and 21 months after birth) using the Edinburgh Postnatal Depression Scale (EPDS; Cox, Holden & Sagovsky, 1987). The EPDS is the most widely used self‐report questionnaire to identify symptoms of depression during the perinatal period. The scale consists of 10 items that describe common symptoms of depression; however, the scale does not include somatic symptoms of depression, such as a change in appetite or fatigue, which are commonly experienced in pregnancy. Each item is scored from 0 to 3, and there is a maximum score of 30. For the measure of maternal postnatal depression, an average of the scores reported at 8 weeks, 8 months and 21 months after birth (all correlated ~.5) was used. The maternal prenatal and postnatal depression measures were square‐root transformed to normalise the distribution. The EPDS was also used to measure prenatal and postnatal depression in the WCHADS cohort.

#### Maternal anxiety

At 18 and 32 weeks of pregnancy, and at 8 weeks, 8 months and 21 months after birth, maternal anxiety was assessed using the anxiety subscale from the Crown Crisp Index, a validated self‐rating inventory (Birtchnell, Evans & Kennard, 1988). The Crown Crisp Index is a 24‐item questionnaire with four subscales, each composed of eight items. Participants were asked to rate their responses to questions relating to feelings and behaviours on a 4‐point scale from ‘very often’ to ‘never’. For the measure of maternal postnatal anxiety, an average of the scores reported at 8 weeks, 8 months and 21 months after birth was used. The maternal prenatal and postnatal anxiety measures were square‐root transformed to normalise the distribution.

#### Child emotional symptoms

Mothers rated child emotional symptoms when the child was aged 3.5 years using the emotional difficulties subscale of the Revised Rutter Scale for Preschool Children (Elander & Rutter, 1996). The scale comprises 43 statements describing child behaviours, and mothers were required to rate the extent to which each item described their child using a 3‐point Likert scale of certainly true, sometimes true and never true. Responses are aggregated to create scores on four domains: emotional difficulties, conduct difficulties, hyperactivity and prosocial behaviour. A square‐root transformation was applied to reduce the skew in the distribution of the emotional difficulties variable. In the WCHADS cohort, child symptoms were assessed by maternal report using the preschool Child Behaviour Checklist (CBCL), which includes an anxious‐depressed subscale (Rescorla et al., 2011).

#### Confounding variables

Mothers reported on the following variables during pregnancy that were included in analyses as confounders: highest educational qualification (binary: degree and above), smoking status at 32 weeks of pregnancy (grouped number of cigarettes per day, in the following categories: none, 1–9, 10–19 and >20), household crowding at 8 weeks of pregnancy (binary: top quintile of crowding index (the number of residents living in a dwelling divided by the number of rooms in a dwelling) and other), maternal age at birth (categorical: <21, 21–30, >30) and relationship status (binary: living with partner and other).

### Statistical analyses

Analyses for the main hypothesis were conducted in accordance with the preregistered analysis plan, (Braithwaite, 2018), ‘Ordinary regression will be used to estimate the emotional difficulties outcome at 3.5 years predicted by the square‐root and standardized transformed pre‐ and postnatal depression scores, and child sex, their product (the three‐way interaction effect) and the main effects of the confounders listed above. Coefficients, 95% confidence intervals and *p*‐values will be reported for the main effects and the interaction effect’.

To account for nonrandom attrition, we used the preregistered method, ‘The association of available prenatal variables for the probability of inclusion by way of complete data will be examined. Those variables that, in the presence of the confounders, show a significant association with inclusion will be factored into quintiles and included in a logistic model to estimate attrition weights, calculated as the inverse of the predicted probability of inclusion. These weights will be used as probability weights in the analyses’.

The two‐way interactions in girls and boys were displayed according to the preregistered plan, ‘Plots of the simple regression line (with 95% confidence interval) of postnatal depression score (X) for emotional problems (Y) will be displayed for participants with high and low prenatal depression (median split), for boys and girls. In addition, and in order to display the sex differences at different levels of prenatal depression, plots of simple regression lines of postnatal depression score for emotional problems contrasting boys and girls at low, medium and high (terciles) levels of prenatal depression were prepared. We also generated postestimation heat (contour) maps from the model fitted in the preregistered analysis to show, marginal to the other variables, the joint contributions of prenatal and postnatal maternal depressive symptoms to emotional symptoms in boys and girls, and to the difference between them. These allow us to show the effects directly from the preregistered analysis, rather than from follow‐up analyses of the two‐way interactions, and to see how risks for child symptoms arise as prenatal and postnatal depression scores change. The different coloured regions demarcate contour lines representing equal steps across the distribution of child emotional problems scores generated from the model.

We conducted planned exploratory analyses with maternal depressive symptoms at 32 weeks gestation instead of 18 weeks, and to examine the three‐way interaction of prenatal by postnatal maternal anxiety by child sex.

The study website http://www.bristol.ac.uk/alspac/researchers/our‐data/ contains details of all the data that are available, through a fully searchable data dictionary and variable search tool.

## Results

Summary statistics for the measures included in the models are displayed in Table 1, split by child sex. Of the 14,853 cases in the ALSPAC cohort, multivariate logistic regression indicated that sample attrition was associated with maternal depression (EPDS) score at 18 weeks gestation, and with maternal depression (EPDS) and anxiety (CCEI) scores at 32 weeks gestation.

**Table 1:** Descriptive statistics for maternal and child variables by child sex

	Male				Female			
	*N*	Mean	*SD*	%	*N*	Mean	*SD*	%
Prenatal depression – 2nd trimester	4,004	6.50	4.58	–	3,773	6.44	4.57	–
Prenatal depression – 3rd trimester	4,001	6.66	4.95	–	3,768	6.56	4.82	–
Postnatal depression – 8 weeks	3,905	5.87	4.60	–	3,690	5.65	4.53	–
Postnatal depression – 8 months	3,865	5.22	4.60	–	3,643	5.10	4.52	–
Postnatal depression – 21 months	4,004	5.53	4.70	–	3,773	5.48	4.71	–
Prenatal anxiety – 2nd trimester	3,997	4.63	3.38	–	3,753	4.65	3.40	–
Prenatal anxiety – 3rd trimester	3,990	4.87	3.50	–	3,748	4.87	3.46	–
Postnatal anxiety – 8 weeks	3,903	3.27	3.19	–	3,688	3.27	3.16	–
Postnatal anxiety – 8 months	3,899	3.53	3.25	–	3,690	3.47	3.23	–
Postnatal anxiety – 21 months	4,004	3.70	3.28	–	3,773	3.70	3.30	–
Maternal age at birth	4,004				3,773			
20 years and younger				2.8				3.1
Between 20 and 30 years				60.8				62.9
Maternal relationship status: living with partner	4,004			95.7	3,773			95.8
Maternal crowding index: top quintile	4,004			4.3	3,773			3.6
Maternal highest educational qualification: degree or above	4,004			14.6	3,773			15.6
Maternal 3rd trimester smoking (any)	4,004			15.6	3,773			15.2
Child emotional symptoms age 3.5 years	4,004	2.47	1.74		3,773	2.55	1.69	

### Examination of the main hypothesis

**Figure 1:**
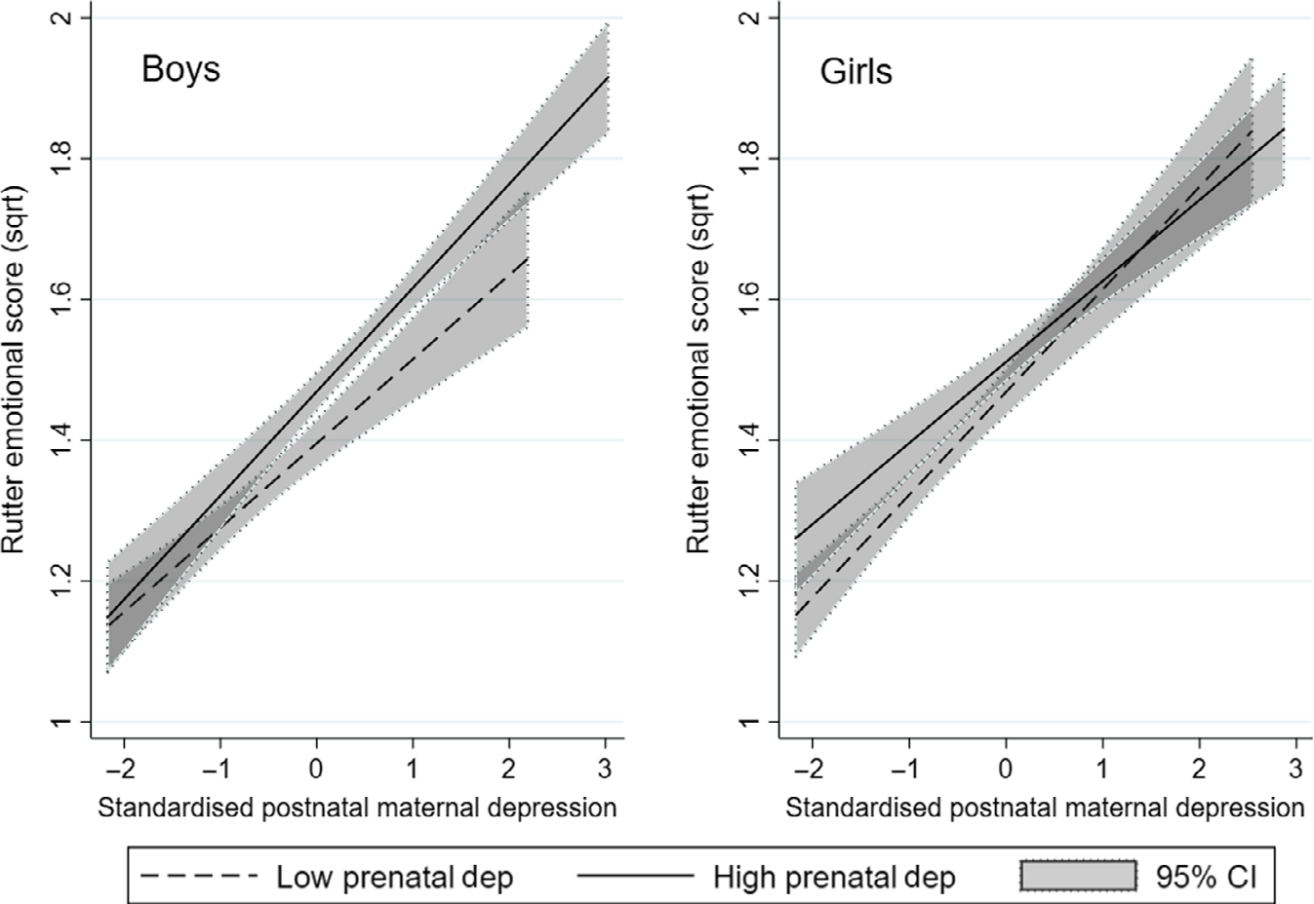
Graphs showing how levels of prenatal depression moderate the association between postnatal maternal depression and child emotional symptoms differently in boys and girls. Plots are shown of the simple regression lines (with 95% confidence interval) of postnatal depression scores for emotional symptoms at age 3.5 years in high and low prenatal depression groups (median split), for boys and girls. In the left hand panel, it can be seen that, in boys, following low levels of maternal depression during pregnancy, postnatal depression is more weakly associated with child symptoms than after elevated prenatal depression. In girls, the pattern is reversed with the stronger association between postnatal maternal depression and child emotional symptoms seen after low prenatal depression (NB in girls the interaction is nonsignificant)

**Figure 2:**
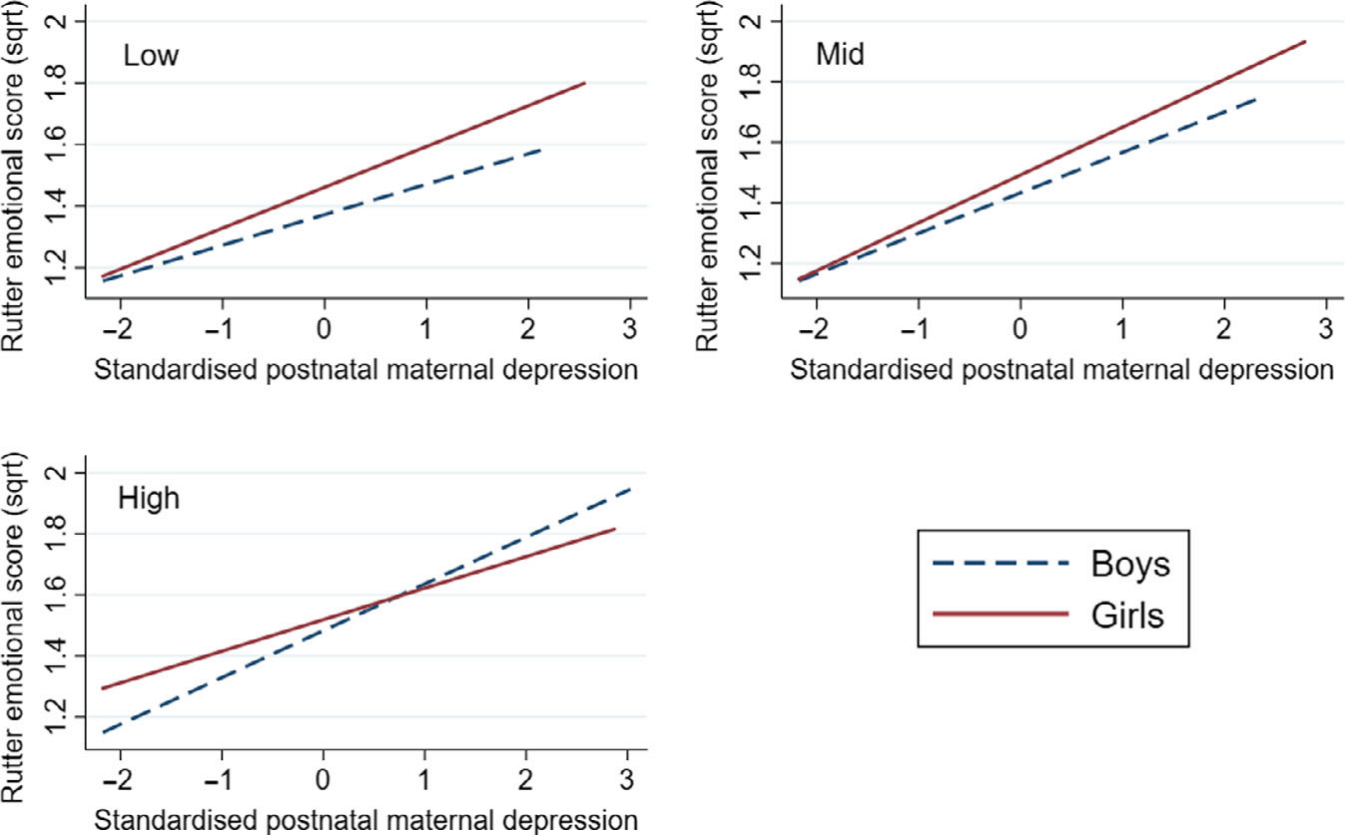
Associations between maternal postnatal depression and child emotional symptoms showing sex differences at three levels of prenatal depression. Plots are shown of the simple regression lines contrasting boys and girls at low, mid and high prenatal depression terciles of prenatal depression scores. In the presence of low prenatal depression, the rate of increase in child emotional symptoms with increasing postnatal depressive symptoms is higher in girls than in boys. It can be seen that girls exposed to the mismatch between prenatal and postnatal conditions (low prenatal depression–high postnatal depression) have higher emotional symptoms than the boys. In the panel showing the sex difference following high prenatal depression, the boys exposed to matched (high prenatal–high postnatal) conditions have higher emotional symptoms than the girls

A regression was fitted to the 8354 complete data cases, weighted for attrition, with the square‐root emotional difficulties subscale of the Rutter Revised Scale at 3.5 years as the outcome variable. The predictors were the main effects, and two‐way and three‐way interactions of the square‐root transformed maternal depression scores at 18 weeks of pregnancy, the average of the three postnatal scores (at 8 weeks, 8 months and 21 months), and a dummy variable for female child. The depression variables were standardised to a mean of zero and variance of one to aid in their interpretation. There was a positive three‐way interaction (.034, 95%CI = 0.009 to 0.060, *p* = 0.008), which arose from the two‐way interactions shown below, and the arbitrary coding of female = 1 and male = 2. The addition of the possible confounding effects of maternal age, education, crowding, partner and smoking status somewhat increased the strength of the three‐way interaction (.042, 95%CI = 0.015 to 0.068, *p* = .002). Figure 1 illustrates the pattern of association represented by the interaction in accordance with the preregistered method. In boys, the slope of the regression line of those exposed to higher prenatal depression was greater than that in the low prenatal depression group and this difference was reflected in a positive two‐way interaction that was statistically significant (coeff = .030, 95% CI = 0.012 to 0.048, *p* = .001). In girls, by contrast, the slope of those exposed to higher prenatal depression was lower, reflected in a negative coefficient, although the test of the two‐way interaction was nonsignificant (coeff = −.012, 95% CI −0.031 to 0.007, *p* = .221). The sex difference is further illustrated in Figure 2, which shows contrasting regression lines for males and females in low, mid and high prenatal depression terciles. It can be seen that in the presence of low prenatal depression the rate of increase in child emotional symptoms with increasing postnatal depressive symptoms was higher in girls than in boys, consistent with the hypothesis that mismatch between prenatal and postnatal conditions (low prenatal depression–high postnatal depression) leads to poorer outcomes in females than in males. By contrast, in the panel showing the sex difference following high prenatal depression, it is the boy exposed to matched (high prenatal–high postnatal) conditions who have the higher emotional symptoms than the girls.

**Figure 3:**
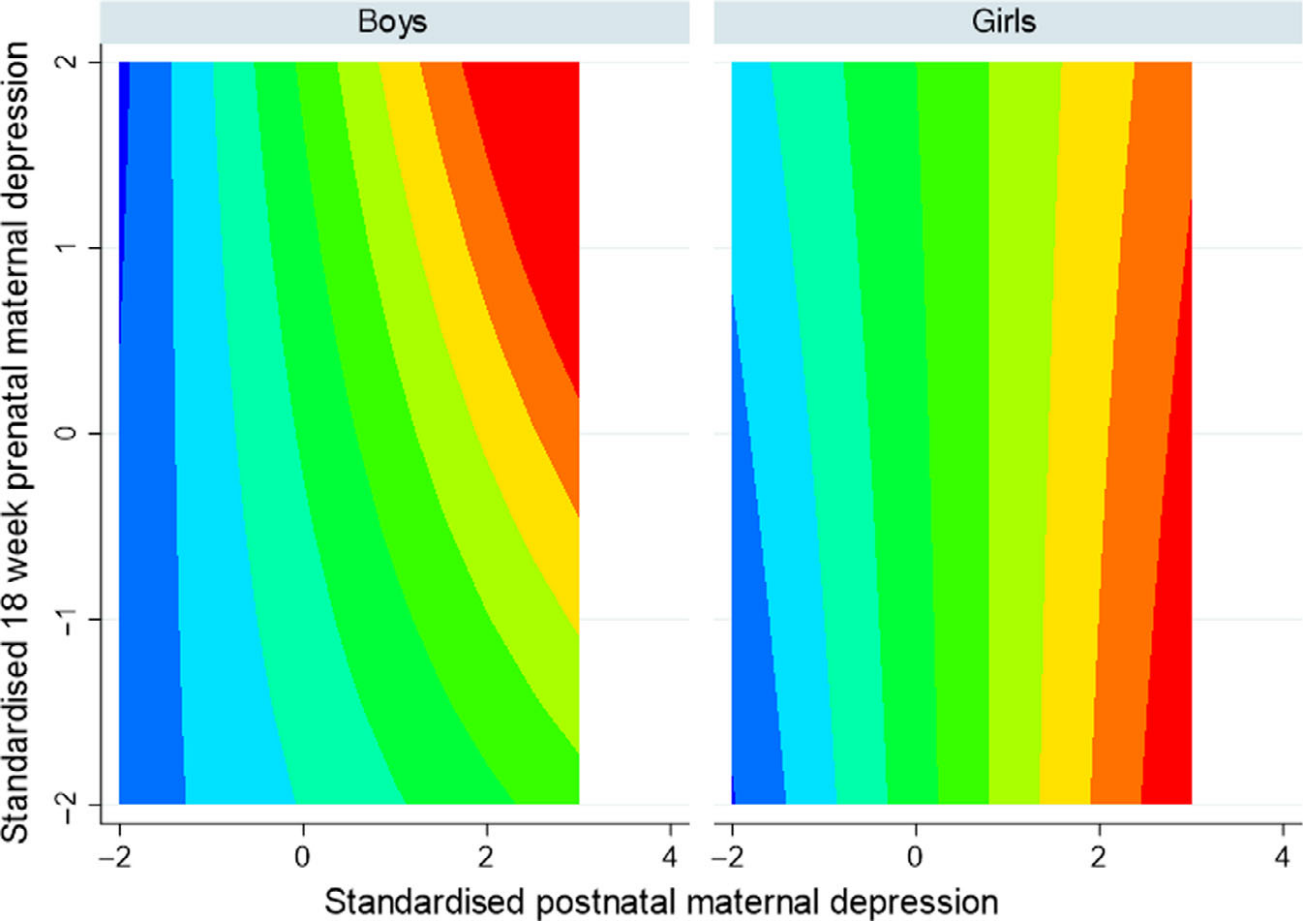
Contour map showing variation in colour intervals of equal sized steps in the level of child emotional problems from high (red) to low (blue) by prenatal and postnatal maternal depressive symptoms for boys and girls. Contour map showing the higher (red) to lower (blue) predicted emotional problem score of girls compared to boys as this difference varies with prenatal and postnatal maternal depressive symptoms

**Figure 4:**
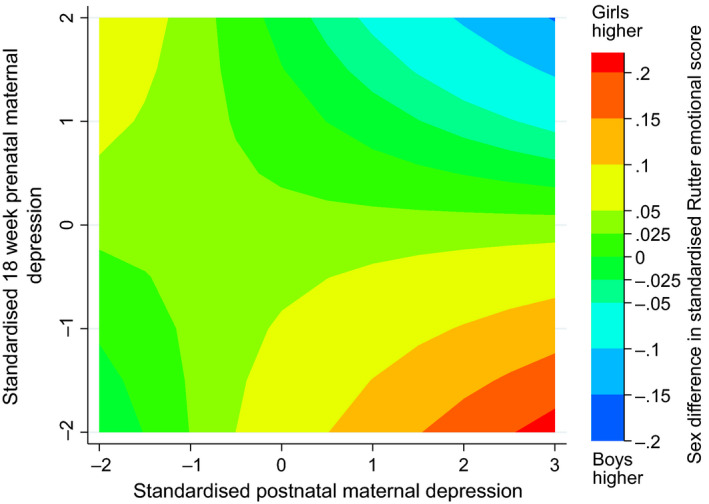
Contour map showing the higher (red) to lower (blue) predicted emotional problem score of girls compared to boys as this difference varies with prenatal and postnatal maternal depressive symptoms

Figures 3 and 4 show, from the model fitted to the whole sample, how levels of child emotional symptoms varied with changes in prenatal and postnatal maternal depression scores. Figure 3 shows that risk of elevated child emotional symptoms associated with maternal postnatal depressive symptoms arose only among those exposed to prenatal depression – the top right of the map for boys. In girls, by contrast high levels of emotional symptoms associated with maternal postnatal depressive symptoms arose more commonly among those not exposed to prenatal depression – the lower right of the map for girls. Figure 4 maps the sex difference in child symptoms and brings out how the mismatched low prenatal depression–high postnatal maternal depression, and to a lesser degree the mismatched high prenatal low postnatal depression, seen in opposite corners of the map, were associated with higher symptoms in girls compared to boys. By contrast, it is the matched high prenatal‐high postnatal depression that was associated with greater emotional symptoms in boys than in girls.

### Exploratory analyses

We did not preregister an examination of whether the predicted sex‐dependent effects would differ according to the timing of postnatal maternal depression. However, we conducted exploratory analyses as a possible basis for future research, which revealed three‐way interactions with maternal depression at each time point: 8 weeks *p* < .001, 8 months *p* = .045, 21 months *p* = .021. Preregistered exploratory analyses of moderation by maternal depressive symptoms at 32 weeks made use of data from the 7,769 cases for which 32 weeks EPDS scores were available and were weighted in the same way for attrition. These gave very similar results with a three‐way prenatal by postnatal by sex of child interaction (.030 95% CI = 0.004 to 0.057, *p* = .026) and a significant two‐way interaction for boys (*p* = .035) and nonsignificant for girls (*p* = .285) with differences in slopes as illustrated in Figure 1. By contrast, equivalent models for anxiety symptoms at 20 and 32 weeks gestation were entirely nonsignificant.

## Discussion

We followed up on previous findings from the Wirral Child Health and Development Study by preregistering replication analyses to be employed with data from ALSPAC, a larger general population longitudinal cohort. We replicated the previous finding that the association between postnatal maternal depressive symptoms and child emotional symptoms is moderated by the level of prenatal depressive symptoms in a sex‐dependent manner. However, in ALSPAC, the sex difference was explained mainly by a protective effect of low prenatal depression in boys, while in WCHADS, it arose from greater vulnerability of girls to postnatal depression following low prenatal depression.

The findings add further support for a phenomenon that we have reported in three previous publications from WCHADS, that prenatal affective symptoms moderate associations between postnatal exposures and child outcomes, assessed as *NR3C1* methylation at age 14 months (Murgatroyd, Quinn, Sharp, Pickles & Hill, 2015), child emotional symptoms up to age 5 years (Hill et al., 2017; Hill, Pickles, Wright, Quinn, et al., 2019) and irritability at age 7 years (Hill, Pickles, Wright, Braithwaite, et al., 2019). In each case, these effects were modified by sex of child. Taken together, the WCHADS and the ALSPAC findings suggest that the combined effects of the PAR and T‐W mechanisms can give rise to outcomes in at least two different ways. In WCHADS, the sex difference was accounted for largely by the vulnerability of girls exposed to the sequence of low prenatal anxiety or depression, followed by high postnatal exposure, which we interpreted as reflecting a lack of the anticipatory effect of high prenatal maternal symptoms. In ALSPAC, as can be seen in the left hand panel of Figure 1, the larger contribution to the sex difference arose from a protective effect of exposure to low maternal depressive symptoms in utero prior to high postnatal symptoms, which we interpret as reflecting the advantages for the male foetus of good maternal conditions during pregnancy in line with T‐W theory. Equally, as shown in the right hand panel of Figure 1, in girls, low prenatal depressive symptoms followed by high postnatal depression were associated with higher child emotional symptoms, as in WCHADS, although this interaction was nonsignificant.

A key strength of the current study is the use of a large, general population cohort, which accounted for a number of plausible confounds and factors associated with attrition. Additionally, we planned, preregistered and tested distinct hypotheses based on our previous research, and we have ensured transparency by also preregistering additional, exploratory analyses. Limitations include that maternal perinatal depression, child emotional symptoms and confounders were all measured by maternal report; therefore, it is possible that common method variance across predictor and outcome variables and biasing effects of maternal mood may have inflated main effects. Maternal mood was not assessed in the ALSPAC study at 3.5 years so we were not able to include it as a covariate in the analyses.

As we described earlier, the hypotheses for this study were derived by considering the joint implications of the predictive adaptive response (PAR) theory and the Trivers–Willard sex biased reproductive investment theories. While these have received relatively little attention in research into human development, they have been extensively investigated in animal studies, where the problems of reporter bias do not arise. Effects consistent with these hypotheses have been shown across many species and environmental conditions, for example in the relationship between sea ice conditions and reproduction in Arctic migratory birds (Jean‐Gagnon et al, 2018), and links between exposure of Salamander eggs to the chemical cues of a predator's presence and subsequent shelter‐seeking behaviour (Mathis, Ferrari, Windel, Messier, & Chivers, 2008). A series of elegant experiments in starlings examined predictions based on the PAR and T‐R hypotheses (Love & Williams, 2008a, 2008b). Prenatal stress was mimicked by injection of corticosterone into starling eggs, and stressful postnatal conditions for chicks were created by wing clipping of mothers after hatching. Corticosterone levels in chicks following a standard stressor were greatest in those who had been exposed to the mismatch condition of no corticosterone injection followed by rearing by wing clipped mothers, and this effect was greater in females than males, as evidenced in a significant sex by matching condition interaction. Furthermore, there was a higher mortality among male chicks from the matched conditions of corticosterone injected eggs reared by wing clipped mothers, significantly shifting the sex ratio in favour of females.

In a test of the implications of the T‐W hypothesis for nutritional variations in humans, Mathews, Johnson and Neil (2008) showed that more males than females were born to mothers with high energy intake during pregnancy, while the sex ratio was reversed in the children of mothers with low energy diets. Support for the predictions of the PAR hypotheses in humans without the limitations of reporting biases is provided by the studies of Sandman and colleagues based on assessments of motor and mental development over the first year of life in infants assessed using the Bayley Scales. The performance of infants exposed to congruent levels of maternal depression, either high prenatal and high postnatal or low prenatal and low postnatal, was higher than those whose mothers had incongruent prenatal and postnatal levels of depression. At 6 months, this PAR effect was confined to female infants, consistent with the T‐W hypothesis, although the sex difference was no longer evident by 12 months (Sandman et al, 2013). As outlined earlier, we have previously shown higher *NR3C1* methylation levels in the children of mothers with the incongruent low prenatal and high postnatal depression than congruent high prenatal–high postnatal levels, and mediation by *NR3C1* methylation of the association with later anxious‐depressed symptoms, in girls only (Hill, Pickles, Wright, Quinn, et al., 2019; Hill, Pickles, Wright, Braithwaite, et al., 2019; Murgatroyd et al, 2015). Further studies with outcomes that are not confounded by reporter effects for example of observed behaviour or biological measures are needed. While we have interpreted the findings within a foetal origins framework which assumes environmental mediation of prenatal and postnatal effects, the design of the study does not allow us to test for competing genetic explanations.

## Conclusion

Apart from the work of Sandman and colleagues, the possibility of sex‐dependent foetal origins effects of maternal mental health in humans has not previously been investigated. However, evidence for sex‐dependent foetal vulnerability to maternal conditions (Aiken & Ozanne, 2013; Meakin, Saif, Jones, Aviles & Clifton, 2017) and for sex‐dependent associations between prenatal anxiety and depression and child outcomes is now substantial (McEwen, 2019; Sutherland & Brunwasser, 2018). Many of these findings have implicated glucocorticoid mechanisms (Braithwaite, Murphy, Ramchandani & Hill, 2017; Buss et al., 2012; Enlow et al., 2018; Graham et al., 2019; Hill et al., 2017). The implications for the investigation of prenatal and postnatal contributions of maternal depression and anxiety are that they need to take account of interdependent as well as independent contributions to risk, and of the likelihood that the processes differ between males and females. Although our predictions drew on the PAR and T‐W hypotheses, it cannot be assumed that the proposed mechanisms account for our findings. However, they do suggest further hypotheses for testing. For example, if the postnatal association is environmentally mediated, we predict sons of mothers with low prenatal anxiety or depression will be protected from effects of postnatal adversities, while daughters will be more vulnerable. There are implications also for stratification of analyses of cohort studies and trials. As a result of moderation by prenatal maternal mental health and by child sex, main effects of risks or treatments that are small or absent may conceal larger, important effects in subgroups.
